# Shifting patterns of natural variation in the nuclear genome of *caenorhabditis elegans*

**DOI:** 10.1186/1471-2148-11-168

**Published:** 2011-06-16

**Authors:** Eleanne Solorzano, Kazufusa Okamoto, Pushpa Datla, Way Sung, RD Bergeron, WK Thomas

**Affiliations:** 1 Hubbard Center for Genome Studies, University of New Hampshire, Durham, NH, US; 2 Department of Molecular, Cellular, and Biomedical Research, University of New Hampshire, Durham, NH, US; 3 Department of Computer Science, University of New Hampshire, Durham, NH, US; 4 Department of Decision Sciences, University of New Hampshire, Durham, NH, US

## Abstract

**Background:**

Genome wide analysis of variation within a species can reveal the evolution of fundamental biological processes such as mutation, recombination, and natural selection. We compare genome wide sequence differences between two independent isolates of the nematode *Caenorhabditis elegans *(CB4856 and CB4858) and the reference genome (N2).

**Results:**

The base substitution pattern when comparing N2 against CB4858 reveals a transition over transversion bias (1.32:1) that is not present in CB4856. In CB4856, there is a significant bias in the direction of base substitution. The frequency of A or T bases in N2 that are G or C bases in CB4856 outnumber the opposite frequencies for transitions as well as transversions. These differences were not observed in the N2/CB4858 comparison. Similarly, we observed a strong bias for deletions over insertions in CB4856 (1.44: 1) that is not present in CB4858. In both CB4856 and CB4858, there is a significant correlation between SNP rate and recombination rate on the autosomes but not on the X chromosome. Furthermore, we identified numerous significant hotspots of variation in the CB4856-N2 comparison.

In both CB4856 and CB4858, based on a measure of the strength of selection (k_a_/k_s_), all the chromosomes are under negative selection and in CB4856, there is no difference in the strength of natural selection in either the autosomes versus X or between any of the chromosomes. By contrast, in CB4858, k_a_/k_s _values are smaller in the autosomes than in the X chromosome. In addition, in CB4858, k_a_/k_s _values differ between chromosomes.

**Conclusions:**

The clear bias of deletions over insertions in CB4856 suggests that either the CB4856 genome is becoming smaller or the N2 genome is getting larger. We hypothesize the hotspots found represent alleles that are shared between CB4856 and CB4858 but not N2. Because the k_a_/k_s _ratio in the X chromosome is higher than the autosomes on average in CB4858, purifying selection is reduced on the X chromosome.

## Background

All variation found in natural populations is ultimately derived from evolutionary forces acting upon mutation and recombination. By studying genome-wide patterns of variation among closely related species, it is possible to make inferences about the evolutionary process of that organism. Although the model organism *Caenorhabditis elegans *has been well studied in biology, our knowledge of the evolution of natural populations of *C. elegans *is limited [1]. In the past, molecular variation in *C. elegans *has been studied using assays involving single mitochondrial and nuclear genes [2], transposons [3,4], amplified fragment length polymorphisms (*aflps*), and microsatellites [5-8].

In this study, we investigate genetic variation in three strains of *C. elegans*: N2, CB4856, and CB4858 using large-scale DNA sequence datasets [9-11]. N2 (from Bristol, England) is the most commonly used lab strain. It is the strain for which the genome sequence has been completely determined. CB4856 is a strain of *C. elegans *from Hawaii while CB4858 is a strain of *C. elegans *from Pasadena, CA. Strains CB4856 and CB4858 were isolated in 1972 and 1971, respectively. Since then, they have been cultured in the lab. In their *C. elegans *population genomics study, Rockman and Kruglyak [12] distinguished 41 haplotypes among 125 wild isolates in their genotype data from 1460 N2-CB4856 SNPs. They indicated that CB4858 shares haplotype 20 with strains from other localities, including AB2-4, from Adelaide, Australia, and CB4855, from Palo Alto, California. The genotypic similarity among CB4858, CB4855, and AB2-4 suggests they may share an ancestor in a laboratory.

Previous analysis of multiple *Caenorhabditis *lines shows extensive variation in nuclear single-nucleotide polymorphism (SNP) density [9], with the Hawaiian strain CB4856 having the highest SNP density and most unique SNPs [13]. Additional polymorphisms in CB4856 were found by Swan et al. [13], and analysis of the distribution of these polymorphisms by Cutter et al. [14] revealed that the polymorphisms occurred more frequently in the arms than in the core in chromosomes one to five, but are randomly distributed in chromosome X.

The patterns of natural variation found in a genome are the result of mutational events that are influenced by both drift and selection. Under neutral theory, a majority of mutations that arise spontaneously are neutral, and their fate (loss or fixation) is primarily determined by drift. In rare cases, a spontaneous mutation will confer a selective advantage (positive selection) or disadvantage (purifying selection). If by chance this mutation is not lost by drift, in consequent generations the mutation will spread throughout the population, and variation at linked sites will either accumulate or be purged. The combination of neutral mutations fixed through drift and the mutations that are accumulated or purged determine the genomic landscape of the organism [15]. By surveying the patterns of natural variation between the closely related *Caenorhabditis *strains CB4856 and CB4858, it is possible to investigate the evolutionary processes of drift and selection occurring in this lineage.

Although previous studies provide insights into *C. elegans *SNP patterns and densities in the natural isolates, a large-scale genomic analysis of the *C. elegans *polymorphic spectrum has not been accomplished until now. Other studies of *C. elegans *have not focused on examining the patterns of variation that we present here, but have instead focused on other important patterns. For example, Weber et al. [16] reported the genome sequence of the LSJ1 (Bristol) strain and compared it to the reference wild type N2 (Bristol). LSJ1 is a sibling of the reference wild type N2. The authors indicated that comparing the genomes of N2 and LSJ1 highlight genetic changes associated with lab domestication and errors in the *C. elegans *reference genome. They confirmed that LSJ1 and N2 are derived from the same Bristol isolate. In addition, they identified 3246 predicted differences between the LSJ1 and N2 genome sequences. Finally, by sequencing two recently isolated wild *C. elegans *strains, CB4856 (Hawaii) and ED3054 (Kenya), they inferred the ancestral state of the original Bristol isolate. All of this makes CB4856 an extremely valuable resource not only for *C. elegans *gene mapping [10] but also for the study of the distribution and spectrum of *Caenorhabditis *SNPs.

Graustein et al. [17] compared the levels of DNA sequence polymorphism among three closely related species in the genus *Caenorhabditis*: two self-fertilizing species, *C. elegans *and *C. briggsae*, and one cross-fertilizing species, *C. remanei*. Jovelin et al. [18] analyzed nucleotide variation within and between species across two olfactory pathways in *Caenorhabditis *to examine the relationship between evolutionary rate and gene function and their position within the pathway. The authors found that transcription factors specifying chemosensory neuron subtype identity exhibit higher levels of nucleotide divergence and polymorphism than the structural genes in the signaling pathways. They found much higher levels of polymorphism within *C. remanei *than within the related species *C. elegans *and *C. briggsae*. Their findings suggest behavioral differences between species in response to olfactory stimuli are likely to involve differences in gene regulation due to the divergence of regulatory genes.

We report the entire spectrum of events critical for understanding the fundamental divergence patterns of these natural isolates. In addition, we compare our results to the N2 MA line results from Denver et al. [19] because we wish to compare the differences in mutation patterns when selection is present (in our study) versus when it is nearly absent (in MA lines).

## Results and Discussion

### Overview of SNP variation

We identified a total of 13360 putative SNPs in our analysis of the CB4856 genome compared to N2. Direct re-sequencing of 55 of these putative SNPs confirmed 51, suggesting our rate of confirmation is 93%. The 55 SNPs chosen for resequencing were representative of all the SNPs in this study. The level of SNP differences between N2 and CB4856 is 28.6 × 10^-4 ^SNP per base with insertion deletion events (indels) contributing slightly more of the variation than base substitutions and inversions (Table 1). This is consistent with the known mutation spectrum described in Denver et al. [19] that reports slightly more indels than SNPs in the *C. elegans *MA lines. In contrast, a recent genome wide comparison of N2 and the strain CB4858 [11] revealed a much lower genome wide SNP level (5.89 × 10^-4 ^SNPs per base) and those differences were dominated by base substitutions. The difference in the ratio of base substitutions to indels in the two comparisons could be attributed to the differences in sequencing approaches used to assay variation. The CB4856 data was based on longer Sanger reads (average trimmed length was 130 bp) while the CB4858 data was based on short read sequencing. Reads with more than one SNP are difficult to assemble when using short read technology. Consequently, in CB4858, reads with more than 2 differences are incompatible with this analysis [11]. In that scenario, sequential or highly clustered SNPs may not be detected in the CB4858 dataset.

**Table 1:** Overview of genome wide differences between N2 and CB4856 or CB4858

Polymorphism	CB4856	CB4856 × 10^-4^ SNPs per base	CB4858	CB4858 × 10^-4^ SNPs per base
**Base**				
**Substitutions**				
**single**	4926	10.6	36568	5.11
**sequential**	476	1.02	564	0.08
**Sub total**	5402	11.6	37132	5.18
**Insertions**	2024	4.34	2668	0.37
**single**				
**sequential**	1197	2.57	0	0.00
**Ins total**	3221	6.91	2668	0.37
**Deletions**	2618	5.62	2393	0.33
**single**				
**sequential**	2009	4.31	0	0.00
**Del total**	4627	9.93	2393	0.33
**Inversions**				
**2 bp**	98	0.21	53	0.01
**3+bp**	12	0.03	23	0.00
**Total Inv**	110	0.24	76	0.01
**Total**	13360	28.7	42269	5.90

The total number of bases assayed in CB4856 was 4658922 (4.6465% of the *C. elegans *reference genome). The numbers of SNPs observed in CB4858 was based on the assay of 77% of the *C. elegans *reference genome (Hiller et al., 2008).

The total number of bases assayed in CB4856 was 4658922 (4.6465% of the *C. elegans *reference genome) while the number of SNPs observed in CB4858 was based on the assay of 77% of the *C. elegans *reference genome [11]. Even though 4.6465% is a small portion of the *C. elegans *reference genome, we assume that the sequences used to obtain the polymorphisms are randomly selected from the entire genome. It is therefore reasonable to compare the results of CB4856 to the results of CB4858.

The base substitution/indel ratio is 0.6883:1 (5402/7848) for CB4856 and 7.3369:1 (37132/5061) for CB4858 (Table 1). Two observations suggest that the differences in the observed base substitution/indel ratios in CB4846 vs CB4858 do not stem from read length differences in the two assays. First, clusters of SNPs (see CB4856 Statistically Significant Hotspots) do not contribute to a large fraction of the total variation. Therefore, sequential indels which may be undetectable using short-read technology do not greatly influence the base substitution/indel ratio. Second, when we limit the comparisons to single base substitutions and indels which are fully assayable using short read technology in CB4858, we find that the discrepancy between these two natural isolates remains (Table 1). Based on the above observations, the CB4856 SNPs have a higher proportion of indels than the CB4858 SNPs. These observations are consistent with those reported by Denver et al. [19] in which *C. elegans *MA lines showed a base substitution/indel ratio of 0.7059:1 (12/17).

### Either the CB4856 genome is getting smaller or the N2 genome is getting larger

Among the SNPs predicted in the comparison of CB4856 and N2, we observed a strong and significant bias for deletions (4627) over insertions (3221). This bias was reflected in single event indels as well as larger indels (see Table 1 and Additional File 1). Although the direction in which the indel occurred is unknown, this pattern differs from the CB4858 ratio (1:1), suggesting two possibilities: (1) the CB4856 genome is becoming smaller or (2) the N2 genome is getting larger because it fails to purge insertions. This result differs from the baseline mutation spectrum in *C. elegans *MA lines in Denver et al. [19]. They observed a high frequency of indels where the mutations appeared to be biased toward insertions. There are two possible explanations for this observation: (1) there is a change in the mutation spectrum between N2 and CB4856 or (2) selection constraints between N2 and CB4856 have changed.

### Sequential vs single SNPs

Sequential SNPs can arise from single mutational events involving multiple base pairs or as a consequence of individual events at adjacent positions. There is a larger proportion of sequential SNPs in CB4856 compared to CB4858 (Table 1). The high proportions of observed sequential base substitutions [3.6% (476/13360)] and indels [24% (3206/13360)] in CB4856 are not consistent with expectations based on a hypothesis of independent adjacent polymorphisms. This suggests a large fraction of the adjacent SNPs result from mutations involving multiple adjacent base pairs or that the adjacent SNPs are not independent. The distribution of the sizes for sequential SNPs, however, suggests that the occurrence of sequential SNPs is negatively correlated with the number of bases involved.

Due to the limitations of short read sequence alignment, the analysis between N2 and CB4858 is limited to sequential base substitutions, indels, and putative inversions with a length of 2 bp. In particular, inversions are a special subclass of sequential changes that occur through a single event. We identified all size classes of inversions in CB4856 and in CB4858 (Additional File 2). These account for only a small fraction [0.82% (110/13360) in CB4856 and 0.18% (76/42269) in CB4858] of all SNPs and do not affect the overall conclusions of our study. We note that in CB4858, the inversions may be downwardly biased due to the CB4858 sequencing method.

### Shifting patterns of Base substitution

The base substitution pattern when comparing N2 against the CB4858 isolate reveals a *C. elegans *transition over transversion bias of 1.32:1 (21145/15993; Table 2). However, when comparing N2 against CB4856, there is no transition over transversion bias (1:1 ratio; Table 2). Furthermore, in CB4856, there is a significant bias in the direction of base substitution. When comparing N2 against CB4856, the frequency of A or T bases in N2 that are G or C bases in CB4856 greatly outnumber the opposite frequencies for transitions as well as transversions. At equilibrium, the numbers of G:C to A:T differences between two genomes should be equal to the number of A:T to G:C differences. The shift in base substitution results in a significant detectable shift in base composition between N2 (35.44%GC) and CB4856 (37.77%GC). In particular, there is a significant difference between the following rates in the N2/CB4856 comparison: G →A lower than A→G; C →T lower than T →C; C →A lower than A→C; G→T lower than T→G. None of these differences were observed in the N2/CB4858 comparison (see Table 3).

**Table 2:** Base substitutions (bs) in CB4856 and CB4858 relative to the N2 reference genome*.

	N2-> CB4856	Base Sub	N2->CB4856	Base Sub	N2>CB4 858	Base Sub	N2->CB4858	Base Sub
**Transitions**								
	G->A	621 (0.11)	A->G	745 (0.14)	G->A	5266 (0.14)	A->G	5375 (0.14)
	C->T	639 (0.12)	T->C	722 (0.13)	C->T	5163 (0.14)	T->C	5341 (0.14)
**Transversions**								
	C->A	278 (0.05)	A->C	374 (0.07)	C->A	1871 (0.05)	A->C	1945 (0.05)
	G->T	276 (0.05)	T->G	345 (0.06)	G->T	1906 (0.05)	T->G	1893 (0.05)
	T->A	480 (0.09)	A->T	484 (0.09)	T->A	3185 (0.09)	A->T	3240 (0.09)
	G->C	240 (0.04)	C->G	232 (0.04)	G->C	964 (0.03)	C->G	989 (0.03)

*Number (Proportions out of all base substitutions in each strain: 5436 base substitutions in CB4856 and 37138 base substitutions in CB4858)

**Table 3:** Bayesian Intervals for difference in rates between different types of transitions and transversions in CB4856 and CB4858.

CB4856 Base sub	N2->CB4856	Base Sub	N2->CB4856	Base Sub	95% Bayesian Interval for difference using MCMC
**CB4856 Transitions **	G->A	621	A->G	745	(-0.031,-0.008)
	C->T	639	T->C	722	(-0.025,-0.003)
					
**CB4856 Transversions **	C->A	278	A->C	374	(-0.024,-0.008)
	G->T	276	T->G	345	(-0.020,-0.005)
	T->A	480	A->T	484	no sign difference
	G->C	240	C->G	232	no sign difference
					
**CB4858 Base sub **	**N2->CB4858 **	**Base Sub **	**N2->CB4858 **	**Base Sub **	**95% Bayesian Interval for difference using MCMC **
					
**CB4858 Transitions **	G->A	5266	A->G	5375	no sign difference
	C->T	5163	T->C	5341	no sign difference
					
**CB4858 Transversions **	C->A	1871	A->C	1945	no sign difference
	G->T	1906	T->G	1893	no sign difference
	T->A	3185	A->T	3240	no sign difference
	G->C	964	C->G	989	no sign difference

The results shown here differ from the expectation of a significant mutation bias from G:C to A:T and G:C to T:A based on mutation accumulation lines [20]. Because the base substitution pattern is consistent with the N2/CB4858 comparison, but different in CB4856, the change is likely either a change in the mutational mechanisms or shifting selective forces. CB4856 is a prime example of base compositional differences (percentage of the CB4856 genome that consists of the various nucleotides A, T, C, G) that can occur within a species.

### CB4856/CB4858 SNP rate of chromosomes

Both CB4856 and CB4858 exhibit significant differences in SNP rates among the chromosomes (Table 4). In particular, chromosomes I to X do not have a uniform SNP rate in either CB4856 (p < 2.2e-16) or CB4858 (p < 2.2e-16). In addition, the SNP rate is higher in the autosomes than in chromosome X in CB4856 (p < 2.2e-16). In CB4856, chromosome III has the smallest SNP rate while chromosome V has the largest SNP rate. Although previous observations of this disparity have been attributed to the mutagenic effect of recombination [21], the random distribution of mutations across all chromosomes in mutation accumulation lines [20] makes it more likely that selective sweeps are purging polymorphisms from different regions of the genome. A comparison of SNP rates between chromosomes in CB4858 is not appropriate because of the stochastic nature of the SNPs found in the CB4858-N2 comparison. There are large stretches of DNA that are shared by N2 and CB4858.

**Table 4:** SNP rate by chromosome in CB4856 and CB4858

	CB4856		CB4858	
Chrome	SNPs	SNP rate	SNPs	SNP rate
I	1752	0.002711	4584	2.98E-04
II	2435	0.003373	16322	0.001047296
				
III	1335	0.00207	8701	0.0006215
IV	2093	0.002544	413	1.9444E-05
V	3620	0.003754	3311	0.000157667
X	2107	0.002406	8868	0.000492667

### CB4856/CB4858 arms versus core SNP rate

We compared the SNP rate in the arms vs the cores in different chromosomes in CB4856 and CB4858 using chi-squared tests. In both strains, 1) the SNP rate in the arms is significantly higher than in the core in all of the chromosomes; 2) the rates of SNPs in the arms are different across the chromosomes; 3) the rates of SNPs in the core are different across the chromosomes (Table 5).

**Table 5:** Comparison of arms vs core SNP rates in CB4856 and CB4858*

SNP rate comparison	CB4856 p-value	CB4858 p-value
overall arms vs core across chromosomes	<2.2e-16	<2.2e-16
arms across chromosomes	<2.2e-16	<2.2e-16
core across chromosomes	<2.2e-16	<2.2e-16
arms vs core in each chromosome		
I	<2.2e-16	<2.2e-16
II	<2.2e-16	<2.2e-16
III	<8.918e-06	<2.2e-16
IV	<2.2e-16	0.5464
V	1.778e-14	2.2e-16

*Based on chi-squared tests

There are many possible reasons why the SNP rates differ between the arms and the core in both CB4856 and CB4858. The first possibility is that there are more genes in the core. Since purifying selection removes mutations from coding regions, if the gene density is higher in the core, fewer SNPs will occur there [9,20,22]. The second possibility is that there may be more recombination away from the core. If recombination is mutagenic [21], higher rates of recombination will increase SNP density. On the other hand, recombination interrupts linkage disequilibrium reducing the efficacy of selection. As stated in Cutter et al. [23], chromosome arms have moderate to high rates of crossover recombination and low gene density and cores have strikingly little recombination despite comprising nearly half of each autosome. Rockman and Kruglyak [12] and Barnes et al. [21] provided evidence of these observations in their studies. Finally, there may be an inherent difference in mutation rates in the arms and the core.

As observed in this paper and by Stein et al. [1], there is a difference in the rate of SNPs in the arms vs the core. Observations by Denver et al [20] have suggested that the baseline rate of mutation in MA lines is not increased in the arms of autosomes. This suggests that selection, not mutation, plays a larger role in the biased physical distribution of polymorphisms. We then conclude that the reason the SNP rate differs in the arms and core regions is primarily due to natural selection.

### Recombination and SNP rate

**Figure 1:**
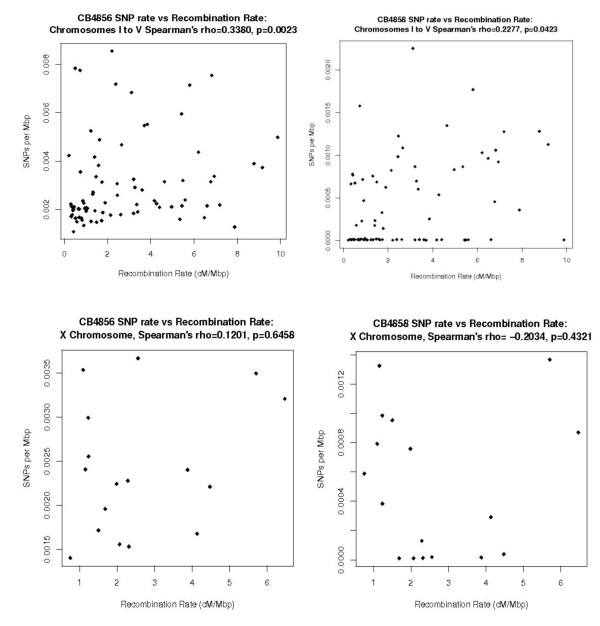
**SNP rates vs. Recombination Rates for CB4856 and CB4858**.

In both CB4856 and CB4858 (Figure 1), the distribution of SNP variation within the *C. elegans *chromosomes shows there is a significant relationship between SNP frequency and recombination rate on the autosomes. However, there is no significant linear relationship between SNP frequency and recombination rate on the X chromosome in either isolate (Table Table 6).

**Table 6:** Relationship between SNP rate and recombination rate in CB4856 and CB4858*

chromosome	CB4856 p-value	CB4858 p-value
autosomes	0.023	0.0423
X chromosome	0.6458	0.4321

*p-values found doing Spearman's rho test for correlation

The discrepancy with the X chromosome can be explained by the X chromosomes' many functional differences from the autosomes [24]. Recombination is almost non-existent in the right terminal arm of the X chromosome [21]. Furthermore, sex is determined by X chromosome dosage and this dosage is compensated in hermaphrodites [25]. Observations of correlation between SNP rate and recombination on the autosomes in both CB4856 and CB4858 have been proposed to either reflect an inherent mutagenic effect of recombination or conditions where selection results in the loss (negative selection) or fixation (positive selection) of new mutations along with all linked polymorphisms. The data in this study shows us that in the autosomes, the observed levels of polymorphisms are positively correlated with levels of recombination in both isolates.

Using the chromosomal domains in Rockman and Kruglyak [12], we found that 1) in the arms, the recombination rate is negatively correlated with chromosome size, although the correlation was not significant (p = 0.303003); 2) the two tips of all autosomes and the X chromosome have an effectively zero recombination; 3) the *C. elegans *autosomes and the X chromosome have nearly constant recombination rates within each chromosomal domain (the slope of the recombination rate over the chromosomal domains is 0). Findings 2) and 3) agree with the findings in Rockman and Kruglyak [12] but finding 1) is different from the finding in Rockman and Kruglyak [12].

### CB4856 Hotspot analysis

The level of SNP variation across the genome in any comparison reflects two possible mechanisms. A high frequency of local polymorphisms can be a consequence of high localized mutation rate or a mutational hotspot. Alternatively, localized patterns of divergence can reflect the age of the allele and historic patterns of recombination. As a consequence of recombination and interbreeding of the ancestors that gave rise to these isolates, each allele can have a unique history of coalescence.

To further investigate the pattern of variation across the *C. elegans *genome and specifically identify regions of significantly high localized polymorphisms, we identified clusters of SNPs with a significant number of polymorphisms (Additional File 3). In general, these hotspots are DNA segments that have an unusual number of polymorphisms considering the length of the DNA (in bp). A more detailed description of the hotspots is given in the Methods section. These hotspots of polymorphisms may be mutational hotspots or may indicate old alleles. We excluded the CB4858 datasets due to the inherent inability of short reads to represent loci with highly clustered polymorphisms.

**Figure 2:**
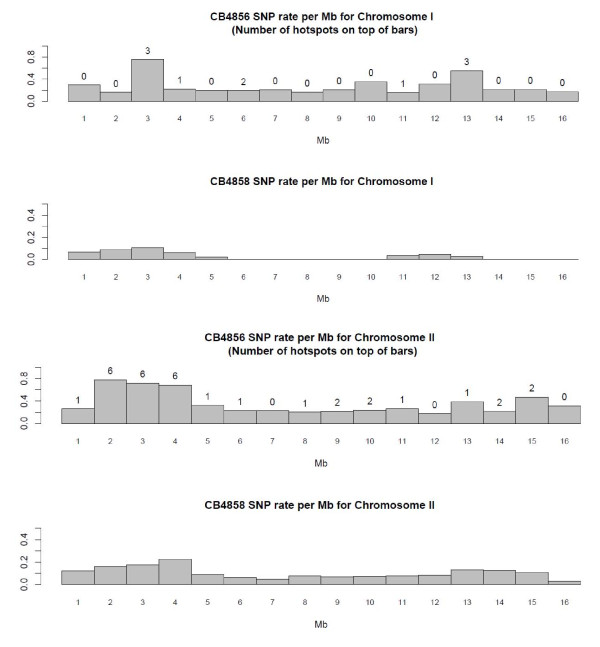
**SNP rate per Mb for Chromosomes I and II in CB4856 and CB4858**.

**Figure 3:**
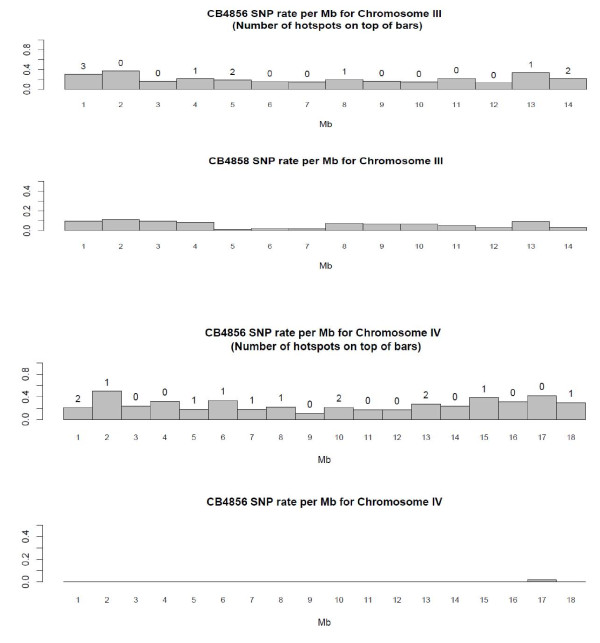
SNP rate per Mb for Chromosomes III and IV in CB4856 and CB4858.

**Figure 4:**
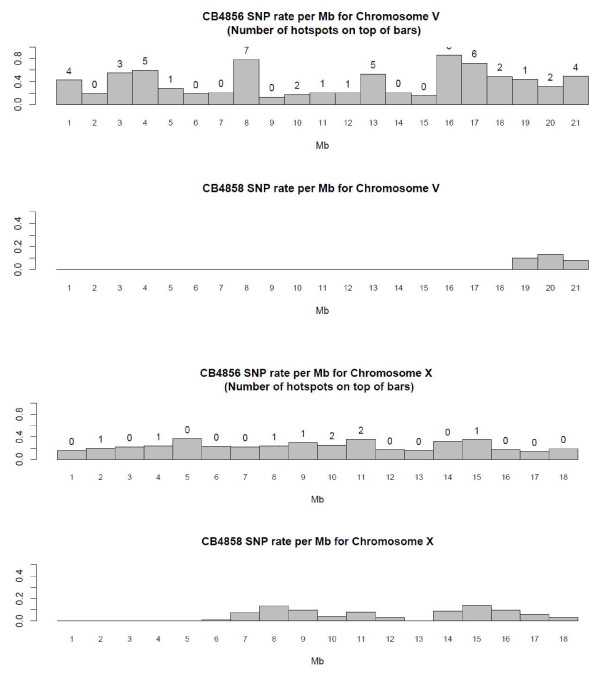
SNP rate per Mb for Chromosomes V and X in CB4856 and CB4858.

The summary statistics of the hotspots per chromosome are shown in Table 7. The optimal window size for the initial cluster analysis was constrained by the average single read length in the CB4856 comparisons (571.14 bp). Statistically significant hotspot clusters ranged from 49 bp to 628 bp in length (Table 8). There were 126 clusters when comparing N2 against CB4856 and the average number of events per hotspot was 17.59. In general, the proportion of base substitutions was the largest in Chromosome V, the proportion of nonsequential indels was the largest in chromosome X, and the proportion of sequential indels was the largest in chromosome IV. There was a significant positive relationship between hotspot length (in bp) and the number of events in the hotspot (p-value = 2.2e-16). Chromosome V has the largest number of hotspots while Chromosome X had the lowest number. We did a linear regression with dummy variables to test if the location of SNPs (arms vs core) was a significant predictor of the number of hotspots. We saw no significant hotspot distributional biases with respect to chromosomal arm/core boundaries (p-value 0.9618, Figures 2, 3 and 4).

**Table 7:** Summary Statistics of CB4856 Statistical Hotspots per Chromosome

CB4856	Hotspots Number of hotspots	Average Number of bases	Average Number of Events	Proportion of Base Substitutions	Proportion of Nonsequential Indels	Proportion of Sequential Indels	Proportion of Inversions
**I**	10	186.1	16	0.47	0.43	0.09	0.01
**II**	32	223.59	18.03	0.54	0.35	0.09	0.01
**III**	10	150.9	11.3	0.36	0.54	0.09	0.01
**IV**	13	180.54	13.08	0.32	0.57	0.11	0.00
**V**	52	292.96	20.67	0.65	0.26	0.09	0.01
**X**	9	145.33	13.56	0.19	0.72	0.08	0.01
**Total**	126	233.44	17.59				

**Table 8:** Distribution of CB4856 Hotspots

	Mean	Standard Deviation	Minimum	Maximum
Length of hotspot (bp)	233.444444	138.2597607	49	628
Number of Events	17.5952381	9.744067792	7	55
Proportion of Base substitutions	0.51654754	0.38545355	0	1
Proportion of non sequential indels	0.38553057	0.381410192	0	1
Proportion of sequential indels	0.08892548	0.095726976	0	0.705882
Proportion of Inversions	0.00899641	0.027289781	0	0.181818

The sizes of the hotspots vary per chromosome. We categorized the hotspots into small and large hotspots based on a cutoff of 100 bp in length. The number of small vs large hotspots is in Table 9. The hotspots with lengths less than 100 base pairs have on average 10.76 of events and 15% of the events are base substitutions while 79% of the events are nonsequential indels. On the other hand, the hotspots with lengths more than 100 base pairs have on average 18.97 events and 58% of the events are base substitutions while 30% of the events are nonsequential indels.

**Table 9:** Total Number of Small and Large Hotspots in CB4856 per Chromosome

Chromosome	Small (<100 bp)	Large (> 100 bp)
I	2	8
II	5	27
III	1	9
IV	3	10
V	8	44
X	2	7

In some taxa, it has been shown that the rate of mutation at specific loci is strongly correlated with GC content [26-31]. To test this possibility, we assayed the GC content in all clusters and compared the %GC content in the clusters to the N2 genome as a whole (%GC).

There is sufficient evidence to indicate the %GC content in the statistical hotspots differs from the %GC content in the N2 genome as a whole (p-value <2.2e-16). The %GC content in different statistical hotspots is displayed in Additional File 4. Some of the hotspots have a higher %GC content than N2 and some of them have a lower %GC content than N2.

We also compared these hotspots of polymorphism to mutations identified in a genome wide analysis of N2 mutation accumulation lines [20]. Denver et al [20] found a random distribution of mutations in their MA lines. The 391 mutations identified among ten N2 derived MA lines did not fall within any of the hotspots identified here. Finally, in a comparison of CB4856 and CB4858, there were 37 SNPs that were shared between CB4856 and CB4858 (Additional File 5), which is larger than the expected number of SNPs shared between CB4856 and CB4858 (approximately 7).

We hypothesize that the hotspots of polymorphisms in this study are alleles that are shared between CB4856 and CB4858 but not by N2 and are not mutational hotspots because (1) the %GC content in the hotspots differs from the %GC content in N2 and (2) because the SNPs found in CB4856 vs N2 were not in the MA lines.

### Distribution of SNPs by function

To investigate the distribution of SNPs across major functional partitions in the *C. elegans *genome, we identified the putative functional role of each SNP position based on the annotation of the N2 reference genome (Table 10). The general variation in both CB4856 and CB4858 follows expected patterns. The functional categories of SNPs are ordered by Intergenic>Introns>UTR>Exons in CB4856 and Intergenic>UTR>Exons>Introns (Table 11). The functional categories of SNPs are ordered as expected for CB4856 but for CB4858, where the frequency of SNPs in exons is higher than in introns. CB4858 may have relaxed selection constraints on coding mutations (smaller population size).

**Table 10:** Frequencies of SNPs by function and chromosome.

	I		II		III		IV		V		X	
	4856	4858	4856	4858	4856	4858	4856	4858	4856	4858	4856	4858
**Function**												
**Exon**												
Insert	9,942	91	16,423	334	10,263	156	11,219	6	8,645	112	11,106	437.11
Deletion	13825.97	78.05	17536.07	320.82	13684.02	192.04	15121.4	5.6	13436	93.73	11907.1	309.85
bs	19107.81	4110.87	41335.02	21232.1	19126.52	11330.5	12926.4	173.73	45618	1944.89	11563.6	6003.35
syn	9165.53	2406.68	19484.52	10292	7775.01	6870.87	5243.72	106.48	23642	876.37	5839.05	3192.57
Total	42876.05	4279.99	75293.76	21886.6	43073.55	11679.1	39267	184.94	67698	2151.1	34576.3	6750.32
**Intron**												
Insert	26409.16	435.8	20319.57	782.81	8397.01	867.75	13536.1	16.81	19268	201.52	14540.4	459.24
Deletion	11806.45	305.71	22268.03	648.06	26590.53	654.37	23657.7	16.81	11769	126.54	15570.8	520.11
bs	19263.15	5281.69	31731.94	14167.6	14306.02	10605	16828.7	173.73	36974	2371.36	14998.3	6877.58
Total	57478.76	6023.21	74319.54	15598.4	49293.56	12127.2	54022.6	207.36	68011	2699.41	45109.5	7856.92
**UTR**												
Insert	11495.75	695.99	21850.5	1803.03	11507.01	1322.96	8536.29	44.83	19789	243.7	7785.4	586.5
Deletion	24,079	852	17,675	1,655	23,325	1,046	15,487	22	16,456	248	18,662	580.97
bs	23457.55	6036.22	25747.41	18421.7	15239.02	11842.7	13658.1	263.4	31558	3791.37	10876.7	7137.63
Total	59032.24	7584.3	65273.15	21880.2	50071.06	14211.2	37681.6	330.65	67802	4283.45	37324.1	8305.1
**IG**												
Insert	23923.59	1144.8	28670.08	3054.24	11351.51	1856.42	27194.2	140.11	38432	328.05	34805.3	1593.52
Deletion	59032.24	787.05	44814.4	2771.91	23791.53	1593.25	58290.7	117.69	53950	313.99	49689.2	1659.91
bs	34021.21	9997.48	54556.66	39538.3	32966.04	20420.6	41827.8	1333.8	84675	5740.94	43277.7	22901.3
Total	116977.1	11929.33	128041.2	45364.4	68109.09	23870.2	127313	1591.6	177057	6382.99	127772	26154.7

The numbers represent isolate × 10^-^^8 ^SNPs per base. *

*For ease in comparison of frequencies across categories and strains, we divided the number of polymorphisms by the number of analyzed sites and multiplied that number by 10^8^. For example, the proportion of insertion polymorphisms in CB4856 is 9942*10^-8^.

**Table 11:** Frequencies SNPs by function in CB4856 and CB4858 across the genome.

CB4856		CB4858	
Type	SNPs	Type	SNPs
Exon	302784.89	Intron	44512.49
UTR	317184.6	Exon	46931.98
Intron	348234.62	UTR	56594.83
IG	745269.1	IG	115293.27

The numbers represent isolate × 10^-^^8 ^SNPs per base.*

*For ease in comparison of frequencies across categories and strains, we divided the number of polymorphisms by the number of analyzed sites and multiplied that number by 10^8^.

### CB4856/CB4858 neutrality

The k_a_/k_s _ratio is the rate of nonsynonymous substitutions (k_a_) to the rate of synonymous substitutions (k_s_), which can be used as an indicator of selective pressure acting on a protein-coding gene. A value of k_a_/k_s_>1 indicates positive selective pressure whereas a value of k_a_/k_s _= 1 indicates neutral evolution. Finally, a value of ka/ks <1 indicates pressures to conserve amino acid sequence [32]. We test for neutrality by testing the null hypothesis of k_a_/k_s _= 1 in both CB4856 and CB4858 [33]. Analysis of genome-wide SNP patterns reveal a k_a_/k_s _of less than 1 for all chromosomes in both CB4856 and CB4858 in Table 12, adjusting for multiple comparisons [34]. This result is consistent with the role that purifying selection has on SNPs in most protein coding regions.

**Table 12:** Mean k-values and p-values testing if ka/ks = 1 in each chromosome*

CB4856			CB4858	
Chrom	ka/ks	p-value for ka/ks = 1	ka/ks	p-value for ka/ks = 1
I	0.41157	0.003471	0.225	1.79E-04
II	0.5746	0.003051	0.3211	3.34E-09
III	0.5496	0.01505	0.2338	2.05E-14
IV	0.5443	0.0115428	0.17435	5.74E-05
V	0.4856	0.00024132	0.3297	1.22E-03
X	0.4261	0.003471	0.2984	4.57E-10

*All ka/ks values in both isolates are significantly smaller than 1

In CB4856, the autosomes have ka/ks values that are not significantly different from X in either direction (p-value = 0.3924). In addition, when considering all of the chromosomes, the ka/ks values are not significantly different in the different chromosomes in either direction (p-value = 0.2343). However, the SNP rate is lower in chromosome X (0.002406) compared to the autosomes (0.00289) in CB4856 (p < 2.2e-16).

Differences in the length or the number of cell divisions in the male germline compared to the hermaphrodites in CB4856 may explain the lower SNP rate in the X chromosome [35]. When the X chromosome is passed through the male germline, it is in a hemizygotic state and exposed to increased purifying selection [35]. In addition, the mitotic cell cycle length has been shown to be shorter in males than hermaphrodite germ cells [36]. Taken together, both of these factors can contribute to a lower SNP rate in the X chromosome than in the autosomes in CB4856. These observations are dependent upon how often males are involved in reproduction from generation to generation. While direct observations of *C. elegans *populations show a low frequency of males and low levels of heterozygosity, it is possible that some lineages of *Caenorhabditis *have historically undergone much more outbreeding involving males [37].

In CB4858, the autosomes have ka/ks values (0.2841) that are significantly smaller than X (0.2984) on the average (p-value = 0.0014). Unlike CB4856, the different chromosomes have significantly different k_a_/k_s _values (p-value = 7.176e-05, Table 13). It is not appropriate to consider the differences in SNP rates between chromosomes in CB4858 due to the long stretches of DNA shared by N2 and CB4858.

**Table 13:** CB4858 chromosomes whose ka/ks values are significantly different and their p-values

Chromosomes	p-value
**II vs IV**	0.0004736
**II vs V**	0.0036938
**III vs IV**	0.0056427
**III vs V**	0.033124
**IV vs X**	0.032036

Because the k_a_/k_s _ratio in the X chromosome is higher than the autosomes on average in CB4858, purifying selection appears to be reduced on the X chromosome in this strain compared to CB4856. The relative difference in ka/ks ratios in CB4858 could reflect differences in the life history of CB4858. We suggest that males (XO) could contribute to a smaller degree in natural populations of CB4858 than in CB4856 populations, resulting in a lower rate of protein sequence evolution for X-linked mutations in the Hawaiian isolate CB4856. Previous studies have presented varying levels of the genetic contribution of males in different natural isolates of *Caenorhabditis elegans *[5,6,38,39].

## Conclusions

This study provides a large scale genomic portrait of the *C. elegans *polymorphisms in CB4856 and CB4858. The patterns observed here give us insight into the evolutionary spectrum of the *Caenorhabditis *lineage. There is a clear bias of deletions over insertions in CB4856, suggesting two possibilities. The first one is that the CB4856 genome is becoming smaller. Other studies have argued that possible reasons that genomes could become smaller in general are gene loss, natural selection, or the occurrence of long terminal repeat (LTR) sequences of transposable elements [40]. The second possibility is that the N2 genome is getting larger because it fails to purge insertions. Interestingly, direct sequencing of *C. elegans *insertion deletion rates in mutation accumulation lines [19] observe a bias towards insertions. Taken together, we conclude that CB4856 is evolving under different mutation or selection pressures than N2 or CB4858.

Our results show that in both CB4856 and CB4858, SNP rates are higher in the chromosome arms than in the chromosome cores in agreement with previous studies [1]. Direct genome sequencing of *C. elegans *mutation accumulation lines [20] show that the mutation rate is not higher in arm regions compared to the core regions. We hypothesize SNP rates differ in the core regions of N2, CB4856, and CB4858 in our study due to higher levels of purifying selection in the core regions.

We found several statistically significant hotspots of polymorphisms in our CB4856-N2 comparison. Some of the CB4856 hotspots contained significantly higher levels of G/C when compared to the genome average, consistent with observations of higher rates of deamination at methylated cytosines [26-28,30,31]. None of these hotspots coincided with *C. elegans *mutations identified in Denver et al. [20], and are likely to be older alleles shared between CB4856 and CB4858 but not N2.

The ka/ks ratios in both CB4856 and CB4858 are consistent with the effect of purifying selection on SNPs in most protein coding regions. The differences in ka/ks in the autosomes vs the X chromosome in CB4858 suggest selection may be more efficient for X-linked genes. In addition, these differences may be attributed to male-driven evolution of the X chromosomes. We hypothesize that natural populations of CB4858 strains have fewer males than CB4856 populations, resulting in a lower rate of protein sequence evolution for X-linked mutations. The finding in CB4858 of a significant difference in selection on the X chromosome compared to the autosomes is consistent with a biological role for males in the lineage giving rise to CB4858, consistent with previous published results [5,6,38,39].

## Methods

### Pipeline for analysis of CB4856 data

The reads for CB4856 were trimmed for quality using the default parameters of LUCY [41], and individually assembled at 90% identity using the AMOS [42] reference assembler against the WS170 *C. elegans *assembly. To reduce the amount of false calls based upon paralogy, each of the polymorphic sites and 70 flanking base pairs was extracted from the reference. These sequences were BLASTed reciprocally at E-5 (~85%) against the genome, and any position that matched multiple sites in the genome was removed from the analysis. The CB4858 base calls followed an identical procedure to remove false polymorphic calls based upon paralogy.

Due to the low coverage of CB4856 reads, it was necessary to set a stringent cutoff of 40 sanger quality score (.0001 base call error rate). CB4856 deletions were scored based on the lowest neighboring quality score. The coding context of both CB4856 and CB4858 polymorphisms were determined using the WS170 General Feature Format (gff).

### Discovery of the statistical hotspots of polymorphisms in CB4856

#### Overall procedure

We did a three step procedure to finalize our statistical hotspots. First, we found initial clusters of SNPs. Then, we decided on an optimal window size to consider for our preliminary hotspots. Finally, we used Gibbs sampling to determine the final statistically significant hotspots.

#### Initial Clusters of SNPs

We initially considered windows of SNPs ranging from 50 bp to 600 bp in increments of 50 bps. For each window, we calculated the mean and standard deviation of the number of SNPs. We then calculated the mean + 3 standard deviations. If there were SNPs outside the window whose location was within 3 standard deviations of the mean, then those SNPs belonged to this cluster. Otherwise, the first SNP which was outside the mean + 3 standard deviations was the beginning of the next cluster. We did this by considering all of the SNPs between N2 and CB4856.

#### Decision on the Size of the Window of the Initial Clusters

A window size of 300 bp yielded the highest number of initial clusters. Assume the number of events in the initial cluster (X) is distributed as Poisson with mean λ = 2.305315 (the average number of events in the 300 bp clusters). To find the total number of events required to be a statistical hotspot, we find number x_o _such that P(X ≥ x_o_) = .0015. This signifies the number of events in the initial cluster (x_o_) is an outlier and a putative hotspot. The number .0015 corresponds to an event which is multiple standard deviations above the mean in our Poisson distribution. The number of events required to be a statistical hotspot (x_o_) in a given bp region is in Additional File 6.

#### Gibbs Sampling Methodology

Gibbs Sampling is an algorithm used to generate a sequence of samples from the joint probability distribution of multiple random variables. It is used as a means of statistical inference. In this work, we used Gibbs Sampling to find posterior confidence intervals to detect statistical hotspots and to detect differences between base substitution rates. Gilks et al. [43] contains a useful description of Gibbs Sampling.

#### Gibbs Sampling Methodology to find Final Statistically Significant Hotspots

Let *X_i _*= total number of events in any 300 bp window i, *θ_i _*= the event rate for window i, and *t_i _*= total number of bp between the first event and the last event in a 300 bp window. Then, *X_i _*~ *Poisson*(*θ_i _t_i _*) for *i = *1,..., *n*, where n is the total number of 300 bp windows being considered. We assume a conjugate gamma prior distribution for the event rate *θ_i _*~ *Gamma*(*α*, *β*) for i = 1, ... n. In addition, we assume the prior specification for the hyperparameters as *α *~ *Exponential*(1.0) and *β *~ *Gamma*(0.08,1.0). We find the posterior distribution of all *θ_i _*| *x_i _*, *α*, *β *for i = 1, ..., n to determine the statistical hotspots and construct 95% credible intervals for the rate of events in a region (λ = θ_i _t_i_). If the lower bound of the 95% credible interval for θ_i_t_i _is above the number x_o _(in Additional File 6), the initial cluster in window i is a statistical hotspot. We do this simultaneously for all initial clusters.

#### Gibbs Sampling Methodology to find the Difference in the Direction of Base Substitution Rates in CB4856-N2 and CB4858-N2 comparisons

The model to test the difference between base substitution rates is described below and is reasonable given the large number of bases assayed in both CB4856 and CB4858. Let θ_j _represent the true difference between base substitution rates and y_j _represent the sample difference between base substitution rates. For example, θ_1 _represents the difference between the G →A and A →G base substitution rates. Then, y_j|_|θ_j_~ Normal  for j = 1,...,6 for the 6 possible base direction changes. The prior distribution θ_j_|μ,τ^2 ^~ Normal (μ, τ^2^). Finally, we have an improper noninformative hyperprior distribution p(μ,τ) α 1. From this, we can obtain the posterior confidence intervals for the direction of base substitution rates given in Table 3. If the posterior confidence intervals do not contain 0, then we conclude there is a difference in the direction of base substitution rates in the isolate considered.

#### Polymorphism Confirmation

Polymorphic loci identified by pairwise comparisons between N2 and CB4856 were selected at random and satisfied the criteria of two or more polymorphic loci within 300-500 bp. Forward primers were designed specific to CB4856. PCR amplification of the 300-500 bp fragments containing the candidate polymorphic base(s) were directly sequenced in the forward direction using standard florescent sequencing technology on an ABI3130 Genetic Analyzer at the University of New Hampshire Hubbard Center for Genome Studies. We found 51 out of 55 polymorphisms were present in the CB4856 genome. The rate of confirmation was 93%.

## Authors' contributions

ES was responsible for the statistical analysis. PD and WS were responsible for the computational techniques necessary to summarize the data. KO was responsible for the biological confirmation of SNPs. RDB was responsible for guiding the bioinformatics aspects of this project. WKT was responsible for guiding the genetics implications of this project. All authors read and approved the final manuscript.

## Supplementary Material

Additional file 1 **Indels in CB4856 compared to N2**. This file contains the Indels in CB4856 vs. N2. Click here for file

Additional file 2 **List of inversions in CB4856 and CB4858**. This file contains the inversions found in CB4856 and CB4858. Click here for file

Additional file 3 **Composition of Hotspots in CB4856**. This file contains the composition of the CB4856 hotspots. Click here for file

Additional file 4 **GC content in different statistical hotspots in CB4856 by chromosome and location**. This file contains the GC content in the CB4856 hotspots by chromosome and location. In N2, the GC content is 35.44%. Click here for file

Additional file 5 **SNPs shared between CB4856 and CB4858 This file contains the SNPs common to CB4856 and CB4858**. Click here for file

Additional file 6 **Number of events required for preliminary cluster to be declared a statistical hotspot of polymorphisms**. This file contains the minimum number of events required for a preliminary CB4856 cluster to be declared a statistical hotspot. Click here for file

## References

[B1] Stein LD Bao Z Blasiar D Blumenthal T Brent MR Chen N Chinwalla A Clarke L Clee C Coghlan A Coulson A D'Eustachio P Fitch DHA Fulton LA Fulton RE Griffiths-Jones S Harris TW Hillier LW Kamath R Kuwabara PE Mardis ER Marra MA Miner TL Minx P Mullikin JC Plumb RW Rogers J Schein JE Sohrmann M Spieth J Stajich JE Wei C Willey D Wilson RK Durbin R Waterston RH The Genome Sequence of *Caenorhabditis briggsae: *A Platform for Comparative Genomics PLoS Biol 2003 1 2 166 192 10.1371/journal.pbio.0000045 PMC261899 14624247

[B2] Thomas WK Wilson AC Mode and tempo of molecular evolution in the nematode Caenorhabditis: cytochrome oxidase I and calmodulin sequences Genetics 1991 128 269 279 1649066 10.1093/genetics/128.2.269 PMC1204465

[B3] Egilmez NK Ebert RH Shmookler Reis RJ Strain evolution in *Caenorhabditis elegans*: transposable elements as markers of interstrain evolutionary history J Mol Evol 1995 40 372 381 10.1007/BF00164023 7769614

[B4] Hodgkin J Doniach T Natural variation and copulatory plug formation in *Caenorhabditis elegans* Genetics 1997 146 149 164 9136008 10.1093/genetics/146.1.149 PMC1207933

[B5] Sivasundar A Hey J Sampling from Natural Populations with RNAi Reveals High Outcrossing and Population Structure in *Caenorhabditis elegans* Current Biology 2005 15 17 1598 1602 10.1016/j.cub.2005.08.034 16139217

[B6] Barriere A Felix M High Local Genetic Diversity and Low Outcrossing Rate in *Caenorhabditis elegans *Natural Populations Current Biology 2005 15 13 1176 1184 10.1016/j.cub.2005.06.022 16005289

[B7] Barriere A Felix M Temporal Dynamics and Linkage Disequilibrium in Natural *C. elegans *Populations Genetics 2007 176 999 1011 17409084 10.1534/genetics.106.067223 PMC1894625

[B8] Cutter AD Félix MA Barrière A Charlesworth D Patterns of nucleotide polymorphism distinguish temperate and tropical wild isolates of Caenorhabditis briggsae Genetics 2006 173 4 2021 31 10.1534/genetics.106.058651 16783011 PMC1569728

[B9] Koch R van Leuenen HG van der Horst M Thijssen KL Plasterk RH Single nucleotide polymorphisms in wild isolates of *Caenorhabditis elegans* Genome Res 2000 10 1690 1696 10.1101/gr.GR-1471R 11076854 PMC310957

[B10] Wicks SR Yeh RT Gish WR Waterston RH Plasterk HA Rapid gene mapping in *Caenorhabditis elegans *using a high density polymorphism map Nature Genetics 2001 28 160 164 10.1038/88878 11381264

[B11] Hillier LW Marth GT Quinlan AR Dooling D Fewell G Barnett D Fox P Glasscock JI Hickenbotham M Huang W Magrini VJ Richt RJ Sander SN Stewart DA Stromberg M Tsung EF Wylie T Schedl T Wilson RK Mardis ER Whole-genome sequencing and variant discovery in *C. elegans* Nature Methods 2008 5 183 188 10.1038/nmeth.1179 18204455

[B12] Rockman MV Kruglyak L Recombinational Landscape and Population Genomics of *Caenorhabditis elegans* PLOS Genetics 2009 5 3 1 16 10.1371/journal.pgen.1000419 PMC2652117 19283065

[B13] Denver DR Lynch M Thomas WK High mutation rate and predominance of insertions in the *Caenorhabditis elegans *nuclear genome Letters to Nature 2004 430 679 682 10.1038/nature02697 15295601

[B14] Swan KA Curtis DE McKusick KB Voinov AV Mapa FA Cancilla MR High-Throughput Gene Mapping in *Caenorhabditis elegans* Genome Research 2002 12 1100 1105 12097347 10.1101/gr.208902 PMC186621

[B15] Cutter AD Payseur BA Selection at Linked Sites in the Partial Selfer *Caenorhabditis elegans* Molecular Biological Evolution 2003 20 5 665 673 10.1093/molbev/msg072 12679551

[B16] Lynch M The Origins of Genomic Architecture 2007 Sunderland MA: Sinauer Associates Inc

[B17] Weber KP De S Kozarewa I Turner DJ Babu MM deBono M Whole Genome Sequencing Highlights Genetic Changes Associated with Laboratory Domestication of *C. elegans* PLOS One 2010 5 11 1 10 10.1371/journal.pone.0013922 PMC2978686 21085631

[B18] Graustein A Gaspar JM Walters JR Palopoli MF Levels of DNA Polymorphism Vary With Mating System in the Nematode Genus *Caenorhabditis* Genetics 2002 161 99 107 12019226 10.1093/genetics/161.1.99 PMC1462083

[B19] Jovelin R Sung FS Phillips PC High Nucleotide Divergence in Developmental Regulatory Genes Contrasts With the Structural Elements of Olfactory Pathways in Caenorhabditis Genetics 181 1387 1397 10.1534/genetics.107.082651 PMC2666507 19001295

[B20] Barnes TM Kohara Y Coulson A Hekimi S Meitic recombination, noncoding DNA and genomic organization in *Caenorhabditis elegans* Genetics 1995 141 1 158 79 10.1093/genetics/141.1.159 PMC1206715 8536965

[B21] Denver DR Dolan PC Wilhelm LJ Sung W Lucas-Lledo JI Hower DK Lewis SC Okamoto K Thomas WK Lynch M Baer CF A genome-wide view of *Caenorhabditis elegans *base-substitution mutation processes PNAS 2009 106 38 16310 163134 10.1073/pnas.0904895106 19805298 PMC2752564

[B22] Denver DR Morris K Thomas WK Phylogenetics in *Caenorhabditis elegans*: An analysis of divergence and outcrossing Mol Biol Evol 2003 20 393 400 10.1093/molbev/msg044 12644560

[B23] Cutter AD Dey A Murray RL Evolution of the *Caenorhabditis elegans *Genome Molecular Biology and Evolution 2009 26 6 1199 1234 10.1093/molbev/msp048 19289596

[B24] Kelly WG Schaner CE Dernburg AF Lee M Kim SK Velleneuve AM Reinke V X-chromosome silencing in the germline of *C. elegans* Development 2002 129 479 492 11807039 10.1242/dev.129.2.479 PMC4066729

[B25] Hodgkin J X chromosome dosage and gene expression in *Caenorhabditis elegans*: Two unusual dumpy genes Mol Gen Genet 1983 192 452 458 10.1007/BF00392190

[B26] Li WH A model for the correlation of mutation rate with GC content and the origin of GC-rich isochors Journal of Molecular Evolution 1994 38 5 468 475 10.1007/BF00178846 8028025

[B27] Fullerton SM Carvalho AB Clark AG Local Rates of Recombination are Positively Correlated with GC Content in the Human Genome Molecular Biol Evol 2001 18 6 1139 1142 10.1093/oxfordjournals.molbev.a003886 11371603

[B28] Filipski J Correlation between molecular clock ticking, codon usage fidelity of DNA repair, chromosome banding and chromatin compactness in germline cells FEBS Lett 1987 217 184 186 10.1016/0014-5793(87)80660-9 3595849

[B29] Sueoka N Directional mutation pressure and neutral molecular evolution Proc Natl Acad Sci 1988 85 2653 2657 10.1073/pnas.85.8.2653 3357886 PMC280056

[B30] Wolfe KH Sharp PM Li WH Mutation rates differ among regions of the mammalian genome Nature 1989 337 283 285 10.1038/337283a0 2911369

[B31] Francino MP Ochman H A comparative genomics approach to DNA asymmetries Ann NY Acad Sci 1999 870 428 431 10.1111/j.1749-6632.1999.tb08919.x 10415514

[B32] Kosakovsky Pond SL Frost SDW Not So Different After All: A Comparison of Methods for Detecting Amino Acid Sites Under Selection Molecular Biology and Evolution 2005 22 5 1208 1222 10.1093/molbev/msi105 15703242

[B33] Zhang Z Li J Zhao X Wang J Wong G Yu J KaKs Calculator: Calculating Ka and Ks Through Model Selection and Model Averaging Genomics, Proteomics & Bioinformatics 2006 4 4 259 263 10.1016/S1672-0229(07)60007-2 17531802 PMC5054075

[B34] Benjamini Y Hochberg Y Controlling the false discovery rate: a practical and powerful approach to multiple testing Journal of the Royal Statistical Society, Series (Methodological) 1995 57 1 289 300

[B35] Vicoso B Charlesworth B Evolution on the *X *chromosome: unusual patterns and processes Nat Rev Genet 2006 7 645 653 10.1038/nrg1914 16847464

[B36] Morgan D Crittenden SL Kimble J The *C. elegans *adult male germline: Stem cells and sexual dimorphism Developmental Biology 2010 346 204 214 10.1016/j.ydbio.2010.07.022 20659446 PMC2945412

[B37] Felix M Braendle C The natural history of *Caenorhabditis elegans* Current Biology 2010 20 R965 R969 10.1016/j.cub.2010.09.050 21093785

[B38] Mayden JS Lorch A Edgley ML Copy number variation in the genomes of twelve natural isolates of *Caenorhabditis elegans* BMC Genomics 2010 11 12 10.1186/1471-2164-11-12 20100350 PMC2822765

[B39] Haber M Schungel M Putz A Muller S Hasert B Schulenburg H Evolutionary History of *Caenorhabditis elegans *Inferred from Microsatellites: Evidence for Spatial and Temporal Genetic Differentiation and the Occurrence of Outbreeding Molecular Biology and Evolution 2005 22 1 160 173 15371529 10.1093/molbev/msh264

[B40] Ridley M Evolution 2003 Malden MA: Wiley-Blackwell

[B41] Chou H Holmes MH DNA sequence quality trimming and vector removal Bioinformatics 2001 17 12 1093 1104 10.1093/bioinformatics/17.12.1093 11751217

[B42] Pop M Philippy A Delcher A Salzberg SL Comparative Genome assembly Briefings in Bioinformatics 2004 5 3 237 248 10.1093/bib/5.3.237 15383210

[B43] Gilks WR Richardson S Spiegelhalter D Markov Chain Monte Carlo in Practice 1995 Boca Raton, FL: Chapman and Hall/CRC

