# Supplementary material for: Shifting patterns of natural variation in the nuclear genome of caenorhabditis elegans
Source: BMC Evol Biol. 2011 Jun 16;11:168. doi: 10.1186/1471-2148-11-168 (PMC3151237; doi:10.1186/1471-2148-11-168)
Supplement: Additional file 1 — Indels in CB4856 compared to N2. This file contains the Indels in CB4856 vs. N2. [file 1471-2148-11-168-S1.PDF]

Additional File 1. Indels in CB4856 compared to N2

base pairs	insertion	deletion
1	2062	2642
2	59	262
3	51	100
4	25	31
5	15	17
6	16	6
7	8	10
8	7	8
9	3	4
10	6	1
11	1	3
12	2	4
13	2	3
14	3	1
15	3	0
16	0	1
17	2	0
18	1	0
19	0	3
20	2	0
21	0	1
22	0	0
23	1	2
24	0	0
25	0	1
26	0	1
27	0	1
28	3	0
29	0	1
30	0	1
31	1	1
32	0	0
33	0	1
34	1	0
38	0	1
42	0	1
44	1	0
58	0	1
72	0	1
75	0	1
	2275	3111
