# Supplementary material for: Shifting patterns of natural variation in the nuclear genome of caenorhabditis elegans
Source: BMC Evol Biol. 2011 Jun 16;11:168. doi: 10.1186/1471-2148-11-168 (PMC3151237; doi:10.1186/1471-2148-11-168)
Supplement: Additional file 2 — List of inversions in CB4856 and CB4858. This file contains the inversions found in CB4856 and CB4858. [file 1471-2148-11-168-S2.PDF]

Additional File 2: List of inversions in CB4856 and CB4858

CB4856

**Chr**	**Position**	**ReadBase**	**SNP**
I	989973	g	c
I	989974	c	g
I	2302923	g	c
I	2302924	c	g
I	4554508	c	g
I	4554509	g	c
I	5047115	g	c
I	5047116	c	g
I	5247151	g	t
I	5247152	t	g
I	6262684	c	g
I	6262685	g	c
I	6511341	g	t
I	6511342	t	g
I	7065003	g	a
I	7065004	a	g
I	9899715	g	t
I	9899716	t	g
I	12364883	c	t
I	12364884	t	c
II	131766	c	a
II	131767	a	c
II	1615556	t	c
II	1615557	c	t
II	1803267	g	a
II	1803268	g	c
II	1803269	c	c
II	1803270	a	g
II	1819990	t	c
II	1819991	g	t
II	1819992	t	t
II	1819993	g	g
II	1819994	c	t
II	2051898	a	g
II	2051899	a	a
II	2051900	a	a
II	2051901	g	a
II	2300602	a	g
II	2300603	a	a

II	2300604	a	a
II	2300605	g	a
II	2695832	t	c
II	2695833	c	t
II	3150306	a	c
II	3150307	a	g
II	3150308	c	a
II	4458820	c	g
II	4458821	g	c
II	4930588	g	t
II	4930589	t	g
II	6857959	a	g
II	6857960	g	a
II	8409572	g	c
II	8409573	c	g
II	8516545	g	c
II	8516546	c	g
II	9994018	t	c
II	9994019	c	t
II	11172837	g	c
II	11172838	c	g
II	11172893	g	c
II	11172894	c	g
II	12017932	g	c
II	12017933	c	g
II	12079683	g	c
II	12079684	c	g
II	12573211	g	c
II	12573212	c	g
II	12932811	g	c
II	12932812	c	g
II	14581682	g	c
II	14581683	c	g
II	14582050	g	c
II	14582051	c	g
III	1998235	g	c
III	1998236	c	g
III	3256473	c	t
III	3256474	t	c
III	4822264	t	a
III	4822265	a	t
III	5462588	a	g
III	5462589	g	a

III	5759054	g	c
III	5759055	c	g
III	5875422	c	g
III	5875423	g	c
III	6413571	t	c
III	6413572	t	t
III	6413573	c	t
III	6830571	g	a
III	6830572	a	a
III	6830573	a	g
III	6841288	g	c
III	6841289	c	g
III	7998202	g	a
III	7998203	a	g
III	8508751	g	t
III	8508752	t	g
III	10021364	g	a
III	10021365	a	g
III	10580591	g	c
III	10580592	c	g
III	12457686	g	c
III	12457687	c	g
III	13220585	c	t
III	13220586	t	c
IV	1055640	g	c
IV	1055641	c	g
IV	3055767	a	g
IV	3055768	g	a
IV	8620064	g	a
IV	8620065	a	g
IV	10962105	c	a
IV	10962106	a	c
IV	12063535	c	g
IV	12063536	g	c
IV	12920105	t	c
IV	12920106	c	t
IV	13019588	c	g
IV	13019589	g	c
IV	15963241	g	c
IV	15963242	c	g
IV	15984681	t	a
IV	15984682	t	t
IV	15984683	a	t

IV	16778871	g	a
IV	16778872	a	g
V	488300	g	c
V	488301	c	g
V	590638	g	c
V	590639	c	g
V	901438	g	c
V	901439	c	g
V	2582405	c	t
V	2582406	t	c
V	3441731	a	g
V	3441732	g	a
V	3785030	t	a
V	3785031	a	t
V	3889730	g	c
V	3889731	c	g
V	4564347	g	a
V	4564348	a	g
V	6334442	c	t
V	6334443	t	t
V	6334444	t	c
V	6344928	c	g
V	6344929	g	c
V	7081009	t	c
V	7081010	t	t
V	7081011	c	t
V	7356619	a	g
V	7356620	g	t
V	7356621	g	g
V	7356622	g	a
V	7602082	c	t
V	7602083	t	t
V	7602084	t	t
V	7602085	t	t
V	7602086	t	c
V	7608327	c	t
V	7608328	t	c
V	9375822	c	t
V	9375823	t	c
V	9982736	g	c
V	9982737	c	g
V	10182439	g	c
V	10182440	c	g

V	10252119	g	c
V	10252120	c	g
V	11821187	g	a
V	11821188	a	g
V	11848040	c	g
V	11848041	g	c
V	12143808	c	g
V	12143809	g	c
V	12188890	g	c
V	12188891	c	g
V	12611823	t	c
V	12611824	c	t
V	12811567	c	g
V	12811568	g	c
V	13670038	g	c
V	13670039	c	g
V	14831066	a	c
V	14831067	c	a
V	15051148	g	c
V	15051149	c	g
V	15161620	g	c
V	15161621	c	g
V	16832966	g	c
V	16832967	c	g
V	19288860	g	a
V	19288861	a	g
V	20128114	c	t
V	20128115	t	c
V	20305922	g	c
V	20305923	c	g
X	3160088	g	c
X	3160089	c	g
X	3972630	g	c
X	3972631	c	g
X	4357410	a	c
X	4357411	c	a
X	5818197	c	g
X	5818198	g	c
X	7255391	g	c
X	7255392	c	g
X	7394569	g	c
X	7394570	c	g
X	7551857	g	c

X	7551858	c	g
X	9757195	a	g
X	9757196	g	a
X	10186272	a	g
X	10186273	g	a
X	10323519	g	c
X	10323520	c	g
X	10927224	a	c
X	10927225	c	a
X	11049448	g	c
X	11049449	c	g
X	11133321	c	g
X	11133322	g	c
X	11841652	g	c
X	11841653	c	g
X	12481795	g	c
X	12481796	c	g
X	13275291	g	c
X	13275292	c	g
X	13356726	c	g
X	13356727	g	c
X	13938973	g	t
X	13938974	t	g
X	14157435	g	c
X	14157436	c	g
X	14353971	g	c
X	14353972	c	g
X	14356831	g	c
X	14356832	c	g

CB4858

**Chr**	**Position**	**ReadBase**	**SNP**
I	6313	G	T
I	6314	T	G
I	1603994	G	A
I	1603995	A	G
I	2152748	A	T
I	2152749	T	A
I	2193789	T	A
I	2193790	T	T
I	2193791	A	T
I	2790905	T	A

I	2790906	A	T
I	3795678	T	G
I	3795679	G	T
I	8362461	C	T
I	8362462	T	C
I	11978689	T	C
I	11978690	C	T
II	911171	C	A
II	911172	A	C
II	1048141	T	C
II	1048142	C	T
II	1252880	G	T
II	1252881	T	G
II	1258325	C	T
II	1258326	T	C
II	1267950	G	A
II	1267951	A	A
II	1267952	A	A
II	1267953	A	A
II	1267954	A	G
II	1288984	A	T
II	1288985	G	G
II	1288986	T	A
II	1294216	T	A
II	1294217	A	A
II	1294218	A	T
II	1615859	T	C
II	1615860	C	C
II	1615861	C	C
II	1615862	C	T
II	1785736	G	T
II	1785737	T	G
II	1789844	A	G
II	1789845	G	A
II	2291967	C	T
II	2291968	T	C
II	2399735	G	A
II	2399736	A	A
II	2399737	A	A
II	2399738	A	G
II	2583814	A	T
II	2583815	T	A
II	3228992	T	G

II	3228993	G	T
II	3245276	G	A
II	3245277	A	G
II	3278318	G	T
II	3278319	T	G
II	3676862	T	C
II	3676863	C	T
II	3722210	C	A
II	3722211	A	C
II	3740977	T	C
II	3740978	C	T
II	3920418	A	T
II	3920419	T	A
II	3920625	G	A
II	3920626	A	A
II	3920627	A	G
II	5681812	A	C
II	5681813	C	A
II	5872506	T	G
II	5872507	G	T
II	9639225	G	T
II	9639226	A	A
II	9639227	T	T
II	9639228	A	A
II	9639229	T	G
II	10549786	G	T
II	10549787	T	T
II	10549788	T	G
II	11232294	G	A
II	11232295	A	G
II	11439722	T	G
II	11439723	G	T
II	11949184	T	C
II	11949185	C	T
II	12210636	A	G
II	12210637	G	G
II	12210638	G	A
II	12295433	A	G
II	12295434	T	T
II	12295435	G	A
II	13418160	C	A
II	13418161	A	C
II	13740080	T	A

II	13740081	A	T
II	14429605	A	T
II	14429606	T	A
III	39346	A	T
III	39347	T	A
III	1307481	A	G
III	1307482	G	A
III	1622411	G	A
III	1622412	T	T
III	1622413	A	G
III	1724303	T	A
III	1724304	A	T
III	2052267	T	A
III	2052268	A	T
III	2370672	A	C
III	2370673	T	T
III	2370674	T	T
III	2370675	C	A
III	2401530	T	G
III	2401531	G	T
III	2528506	T	G
III	2528507	G	G
III	2528508	G	G
III	2528509	G	T
III	2982171	T	C
III	2982172	C	C
III	2982173	C	C
III	2982174	C	T
III	3111402	A	T
III	3111403	T	A
III	6707293	T	A
III	6707294	A	T
III	8733295	T	A
III	8733296	A	T
III	9736613	T	A
III	9736614	A	T
V	18245984	G	T
V	18245985	C	C
V	18245986	T	G
V	18711847	T	A
V	18711848	A	T
V	18738503	T	A
V	18738504	A	A

V	18738505	A	T
V	18754534	G	A
V	18754535	C	C
V	18754536	A	G
V	18754616	G	A
V	18754617	A	G
V	19365031	C	T
V	19365032	T	T
V	19365033	T	C
V	19382267	G	A
V	19382268	A	G
V	20010813	T	C
V	20010814	T	T
V	20010815	T	T
V	20010816	T	T
V	20010817	C	T
V	20138561	C	G
V	20138562	T	T
V	20138563	G	C
V	20215409	A	G
V	20215410	G	A
X	6137437	A	G
X	6137438	G	A
X	6893309	T	A
X	6893310	A	T
X	7404888	T	A
X	7404889	G	G
X	7404890	A	A
X	7404891	G	G
X	7404892	A	T
X	7421301	T	G
X	7421302	A	A
X	7421303	A	A
X	7421304	G	T
X	10114448	T	A
X	10114449	A	T
X	10221235	T	A
X	10221236	A	T
X	10595311	G	A
X	10595312	A	G
X	13136735	A	C
X	13136736	C	A
X	14347733	C	A

X	14347734	A	C
X	14489587	G	T
X	14489588	T	G
X	15787386	T	A
X	15787387	A	T
X	17501939	G	T
X	17501940	T	G
