# Supplementary material for: Shifting patterns of natural variation in the nuclear genome of caenorhabditis elegans
Source: BMC Evol Biol. 2011 Jun 16;11:168. doi: 10.1186/1471-2148-11-168 (PMC3151237; doi:10.1186/1471-2148-11-168)
Supplement: Additional file 3 — Composition of Hotspots in CB4856. This file contains the composition of the CB4856 hotspots. [file 1471-2148-11-168-S3.PDF]

Additional File 3: Composition of Hotspots in CB4856

chr	start	stop	length	events	% baseSub	%nonSeqIndel	% seqIndel	% inversion
I	2150138	2150266	128	11	1	0	0	0
I	2693439	2693974	535	51	0.941176471	0	0.058823529	0
I	2800493	2800655	162	14	0.714285714	0	0.285714286	0
I	3815148	3815281	132.99	13	0	0.923076923	0.076923077	0
I	5757189	5757321	132.01	11	0	0.909090909	0.090909091	0
I	5965005	5965091	85.99	9	0.444444444	0.555555556	0	0
I	10692813	10692892	79.01	11	0	1	0	0
I	12104970	12105236	266	19	0.684210526	0.157894737	0.157894737	0
I	12364883	12365027	144	10	0.1	0.8	0	0.1
I	12982908	12983104	196	11	0.818181818	0	0.181818182	0
II	912335	912631	296	19	1	0	0	0
II	1531786	1532134	348	22	0.636363636	0.136363636	0.227272727	0
II	1608933	1609048	115.01	10	0.9	0.1	0	0
II	1615407	1615637	230	40	0.925	0.025	0.025	0.025
II	1758176	1758542	366	20	0.6	0.1	0.3	0
II	1790243	1790475	232	18	0.666666667	0.111111111	0.222222222	0
II	1819925	1820147	222	11	0.636363636	0.090909091	0.090909091	0.181818182
II	2025858	2025990	132	12	1	0	0	0
II	2051582	2051901	319	40	0.75	0.1	0.1	0.05
II	2058280	2058573	293	12	0.583333333	0.25	0.166666667	0
II	2177219	2177465	246	16	0.6875	0.125	0.1875	0
II	2300455	2300742	287	22	0.909090909	0	0	0.090909091
II	2418945	2419041	96	11	0.818181818	0.181818182	0	0
II	3149965	3150308	342.99	37	0.810810811	0.081081081	0.081081081	0.027027027
II	3367008	3367337	328.99	20	0.75	0.15	0.1	0
II	3401251	3401498	247	15	0.933333333	0	0.066666667	0
II	3673016	3673170	154	17	0.823529412	0.058823529	0.117647059	0
II	3673599	3673866	267	21	0.761904762	0.19047619	0.047619048	0
II	3758831	3759360	529	23	0.869565217	0.086956522	0.043478261	0
II	4855674	4855736	62	9	0	1	0	0
II	5104099	5104261	161.99	24	0.041666667	0.791666667	0.166666667	0
II	7847332	7847424	92	19	0	0.947368421	0.052631579	0
II	8523155	8523235	80.01	9	0	0.888888889	0.111111111	0
II	8721338	8721500	162	10	0	0.8	0.2	0
II	9326081	9326216	135.01	15	0	0.933333333	0.066666667	0
II	9993915	9994019	103.99	10	0	0.8	0.1	0.1
II	10758799	10758910	111.01	13	0.076923077	0.846153846	0.076923077	0
II	12522752	12523244	491.99	24	0.791666667	0.125	0.083333333	0
II	13320664	13320806	141.99	10	0.2	0.8	0	0

II	13483891	13484091	200.01	12	0.083333333	0.833333333	0.083333333	0
II	14232212	14232278	66	15	0.8	0.133333333	0.066666667	0
II	14426574	14426870	296	21	0.238095238	0.666666667	0.095238095	0
III	15892	16052	160	10	0.6	0.1	0.3	0
III	203381	203509	128	10	0.8	0.2	0	0
III	910036	910188	152	10	0	0.8	0.2	0
III	3484646	3484774	127.99	9	0	0.888888889	0.111111111	0
III	4822068	4822294	226	11	0.727272727	0.181818182	0	0.090909091
III	4943361	4943415	54	9	0	1	0	0
III	7639329	7639484	155	10	0	0.9	0.1	0
III	12229396	12229549	153	9	0.333333333	0.444444444	0.222222222	0
III	13363804	13363959	155	10	0.1	0.9	0	0
III	13400925	13401123	198	25	1	0	0	0
IV	833970.01	834159	188.99	16	0.8125	0.125	0.0625	0
IV	972302.01	972517	214.99	11	0.363636364	0.272727273	0.363636364	0
IV	1349510	1349743	233.01	14	0.714285714	0.285714286	0	0
IV	4855202	4855365	163	16	0	0.875	0.125	0
IV	5270670	5270744	74	15	0.066666667	0.933333333	0	0
IV	6926506	6926772	266.05	14	0.142857143	0.714285714	0.142857143	0
IV	7459004	7459383	379	19	0.947368421	0	0.052631579	0
IV	9302377	9302500	123.01	11	0	0.727272727	0.272727273	0
IV	9453460	9453557	97.01	9	0	0.888888889	0.111111111	0
IV	12717589	12717639	50	7	0.142857143	0.857142857	0	0
IV	12830630	12830870	240	16	0.9375	0.0625	0	0
IV	14884157	14884311	154	11	0	0.909090909	0.090909091	0
IV	17374274	17374438	163.99	11	0	0.818181818	0.181818182	0
V	388191.01	388246	54.99	8	0.125	0.875	0	0
V	539419	539749	330	20	0.85	0.05	0.1	0
V	563527.01	563774	246.99	23	0.695652174	0.217391304	0.086956522	0
V	579247.03	579680	432.97	20	0.8	0.15	0.05	0
V	2582388	2582733	345	26	0.692307692	0.153846154	0.115384615	0.038461538
V	2811462	2811729	267	22	0.954545455	0	0.045454545	0
V	2815545	2815931	386	24	0.708333333	0.208333333	0.083333333	0
V	3441488	3441833	345	16	0.625	0	0.3125	0.0625
V	3445299	3445438	139	10	0.9	0	0.1	0
V	3910225	3910432	207	10	1	0	0	0
V	3911253	3911727	474	50	0.86	0.1	0.04	0
V	3950869	3951298	429	28	0.785714286	0.178571429	0.035714286	0
V	4038857	4039082	225	13	0.769230769	0.153846154	0.076923077	0
V	7353876	7354249	373	21	0.857142857	0.095238095	0.047619048	0
V	7356262	7356788	526	50	0.8	0.06	0.1	0.04
V	7595592	7595789	197	15	0.8	0.066666667	0.133333333	0
V	7601873	7602385	512	30	0.766666667	0.033333333	0.133333333	0.066666667

V	7603205	7603569	363.99	23	0.826086957	0.086956522	0.086956522	0
V	7607833	7608350	517	30	0.933333333	0	0.033333333	0.033333333
V	7610529	7610799	270	13	1	0	0	0
V	9672275	9672453	178.01	14	0.071428571	0.857142857	0.071428571	0
V	9803877	9803981	103.99	9	0	0.888888889	0.111111111	0
V	10728878	10728927	49	9	0	1	0	0
V	11774636	11774736	99.99	9	0	0.888888889	0.111111111	0
V	12443712	12443999	287	21	0.80952381	0.095238095	0.095238095	0
V	12446122	12446750	628	55	0.981818182	0	0.018181818	0
V	12636644	12636780	136	9	0.777777778	0.222222222	0	0
V	12700647	12700777	130	11	0.090909091	0.818181818	0.090909091	0
V	12995049	12995099	50	8	0	0.875	0.125	0
V	15378434	15378525	91.01	8	0.25	0.75	0	0
V	15550780	15550859	79	12	0.083333333	0.833333333	0.083333333	0
V	15612060	15612146	86	17	0	0.941176471	0.058823529	0
V	15727872	15728310	438	31	0.612903226	0.290322581	0.096774194	0
V	15735484	15735640	156	11	0.909090909	0	0.090909091	0
V	15936335	15936741	406	37	0.783783784	0.081081081	0.135135135	0
V	15937089	15937643	554.02	33	0.96969697	0	0.03030303	0
V	15955402	15955720	318	23	0.826086957	0.086956522	0.086956522	0
V	16175453	16175875	422	23	0.913043478	0.043478261	0.043478261	0
V	16216490	16216899	409	38	0.894736842	0.105263158	0	0
V	16238646	16238936	290	17	0.235294118	0.058823529	0.705882353	0
V	16595841	16596096	255.01	14	0.714285714	0.142857143	0.142857143	0
V	16809543	16809655	112.01	12	0.166666667	0.75	0.083333333	0
V	16845104	16845198	93.99	9	0	0.888888889	0.111111111	0
V	17294185	17294768	583	30	0.866666667	0.066666667	0.066666667	0
V	17319766	17320082	316	22	0.954545455	0	0.045454545	0
V	18242451	18242940	489	37	0.864864865	0.027027027	0.108108108	0
V	19288730	19288919	189	15	0.933333333	0	0	0.066666667
V	19511919	19512205	286	14	1	0	0	0
V	20128114	20128325	211	13	0.692307692	0.076923077	0.153846154	0.076923077
V	20134353	20134653	300	19	0.631578947	0.210526316	0.157894737	0
V	20570634	20571154	520	24	1	0	0	0
V	20606034	20606361	327	19	0.894736842	0.052631579	0.052631579	0
X	1555981	1556234	253	13	0.230769231	0.692307692	0.076923077	0
X	3897694	3897806	112.01	15	0	1	0	0
X	7865806	7865970	164	17	0.058823529	0.882352941	0.058823529	0
X	8393083	8393246	163	17	0	0.823529412	0.176470588	0
X	9221647	9221787	139.99	10	0	0.8	0.2	0
X	9269725	9269864	139.01	13	0.076923077	0.846153846	0.076923077	0
X	10186239	10186326	86.99	12	0.5	0.416666667	0	0.083333333
X	10524018	10524094	76	11	0	0.909090909	0.090909091	0

X	14221194	14221368	174	14	0.857142857	0.142857143	0	0
