# Supplementary material for: Shifting patterns of natural variation in the nuclear genome of caenorhabditis elegans
Source: BMC Evol Biol. 2011 Jun 16;11:168. doi: 10.1186/1471-2148-11-168 (PMC3151237; doi:10.1186/1471-2148-11-168)
Supplement: Additional file 4 — GC content in different statistical hotspots in CB4856 by chromosome and location. This file contains the GC content in the CB4856 hotspots by chromosome and location. In N2, the GC content is 35.44%. [file 1471-2148-11-168-S4.PDF]

Additional File 4: GC content in different statistical hotspots in CB4856 by chromosome and location. In N2, the GC content is 35.44%.

Chromosome	%GC
I_10692813_10692892	27.8481
I_12104970_12105236	36.09023
I_12364883_12365027	41.66667
I_12982908_12983104	42.34694
I_2150138_2150266	45.3125
I_2693439_2693974	42.61682
I_2800493_2800655	44.44444
I_3815149_3815281	43.93939
I_5757189_5757321	40.15152
I_5965006_5965091	45.88235
II_10758799_10758910	47.74775
II_12522753_12523244	37.88187
II_13320665_13320806	49.64539
II_13483891_13484091	48.5
II_14232212_14232278	33.33333
II_14426574_14426870	32.43243
II_1531786_1532134	32.18391
II_1608933_1609048	44.34783
II_1615407_1615637	39.13043
II_1758176_1758542	38.25137
II_1790243_1790475	38.7931
II_1819925_1820147	34.23423
II_2025858_2025990	47.72727
II_2051582_2051901	38.87147
II_2058280_2058573	33.4471
II_2177219_2177465	38.61789
II_2300455_2300742	37.63066
II_2418945_2419041	48.95833
II_3149966_3150308	35.08772
II_3367009_3367337	36.58537
II_3401251_3401498	35.22267
II_3673016_3673170	43.50649
II_3673599_3673866	36.70412
II_3758831_3759360	34.59357
II_4855675_4855736	34.42623
II_5104100_5104261	43.47826
II_7847333_7847424	37.36264
II_8523155_8523235	37.5

II_8721338_8721500	33.95062
II_912335_912631	43.24324
II_9326081_9326216	27.40741
II_9993916_9994019	39.80583
III_12229396_12229549	45.09804
III_13363804_13363959	31.6129
III_13400925_13401123	32.32323
III_15892_16052	27.5
III_203381_203509	35.9375
III_3484647_3484774	40.15748
III_4822068_4822294	41.15044
III_4943362_4943415	39.62264
III_7639329_7639484	41.93548
III_910036_910188	42.10526
IV_12717590_12717639	38.77551
IV_12830630_12830870	23.75
IV_1349510_1349743	36.0515
IV_14884157_14884311	31.81818
IV_17374275_17374438	43.55828
IV_4855202_4855365	36.80982
IV_5270671_5270744	30.13699
IV_6926506_6926772	29.69925
IV_7459004_7459383	31.13456
IV_833971_834159	27.65957
IV_9302377_9302500	33.33333
IV_9453460_9453557	30.92784
IV_972303_972517	48.59813
V_10728879_10728927	41.66667
V_11774637_11774736	32.32323
V_12443712_12443999	32.05575
V_12446122_12446750	32.64331
V_12636644_12636780	30.14706
V_12700647_12700777	40
V_12995050_12995099	26.53061
V_15378434_15378525	37.36264
V_15550781_15550859	30.76923
V_15612061_15612146	30.58824
V_15727872_15728310	46.11872
V_15735484_15735640	39.74359
V_15936335_15936741	36.2069
V_15937089_15937643	29.9639
V_15955402_15955720	30.18868
V_16175453_16175875	30.09479

V_16216490_16216899	33.98533
V_16238646_16238936	34.48276
V_16595841_16596096	32.15686
V_16809543_16809655	25
V_16845105_16845198	40.86022
V_17294185_17294768	36.36364
V_17319766_17320082	40.82278
V_18242451_18242940	38.44581
V_19288730_19288919	39.15344
V_19511919_19512205	47.55245
V_20128114_20128325	17.53555
V_20134353_20134653	25.66667
V_20570634_20571154	41.34615
V_20606034_20606361	44.0367
V_2582388_2582733	40.86957
V_2811462_2811729	40.82397
V_2815545_2815931	35.23316
V_3441488_3441833	30.14493
V_3445299_3445438	35.2518
V_388192_388246	25.92593
V_3910225_3910432	32.36715
V_3911253_3911727	29.74684
V_3950869_3951298	34.49883
V_4038857_4039082	32.44444
V_539419_539749	31.81818
V_563528_563774	30.89431
V_579248_579680	36.11111
V_7353876_7354249	31.0992
V_7356262_7356788	27.94677
V_7595592_7595789	36.54822
V_7601873_7602385	29.88281
V_7603206_7603569	42.69972
V_7607833_7608350	37.13733
V_7610529_7610799	35.55556
V_9672275_9672453	39.88764
V_9803878_9803981	28.15534
X_10186240_10186326	37.2093
X_10524019_10524094	34.66667
X_14221194_14221368	40.22989
X_1555981_1556234	41.50198
X_3897694_3897806	27.67857
X_7865806_7865970	31.70732
X_8393083_8393246	29.44785

X_9221648_9221787	38.1295
X_9269725_9269864	43.16547
