# Supplementary material for: Shifting patterns of natural variation in the nuclear genome of caenorhabditis elegans
Source: BMC Evol Biol. 2011 Jun 16;11:168. doi: 10.1186/1471-2148-11-168 (PMC3151237; doi:10.1186/1471-2148-11-168)
Supplement: Additional file 6 — Number of events required for preliminary cluster to be declared a statistical hotspot of polymorphisms. This file contains the minimum number of events required for a preliminary CB4856 cluster to be declared a statistical hotspot. [file 1471-2148-11-168-S6.PDF]

Additional File 6: Number of events required for preliminary cluster to be declared a statistical hotspot of polymoprhisms

bp in preliminary clusters	events required
4	2
5	2
6	2
7	2
8	2
9	2
10	2
11	2
12	2
13	2
14	2
15	2
16	2
17	2
18	2
19	3
20	3
21	3
22	3
23	3
24	3
25	3
26	3
27	3
28	3
29	3
30	3
31	3
32	3
33	3
34	3
35	3
36	3
37	3
38	3
39	3
40	3
41	3
42	3

43	3
44	3
45	3
46	3
47	3
48	3
49	3
50	3
51	3
52	3
53	3
54	3
55	3
56	4
57	4
58	4
59	4
60	4
61	4
62	4
63	4
64	4
65	4
66	4
67	4
68	4
69	4
70	4
71	4
72	4
73	4
74	4
75	4
76	4
77	4
78	4
79	4
80	4
81	4
82	4
83	4
84	4
85	4

86	4
87	4
88	4
89	4
90	4
91	4
92	4
93	4
94	4
95	4
96	4
97	4
98	4
99	4
100	4
